# Emphasizing Patient-Centricity Through a Tailored Training Program to Empower Patients, Advocates, and Ethics Committees in Good Clinical Practice

**DOI:** 10.7759/cureus.64042

**Published:** 2024-07-07

**Authors:** Poonam Bagai, Pooja Sharma, Aala Ansari, Nirbhay Singh, Sonal Sharma, Padam Singh, Durga Chougule, Manish Kumar Singh, Gargi Singh, Sanjeev Singh

**Affiliations:** 1 Pediatric Oncology, CanKids KidsCan, New Delhi, IND; 2 Obstetrics and Gynecology, APAR Health, Gurugram, IND; 3 Pediatric Cancer Research, CanKids KidsCan, New Delhi, IND; 4 Patient Navigation, Advocacy, and Family Engagement, CanKids KidsCan, New Delhi, IND; 5 Clinical Research, Medanta Institute of Education and Research, Gurgaon, IND; 6 Amrita Institute of Medical Sciences, Amrita Hospital, Faridabad, IND

**Keywords:** ethics committee, patient navigators, patient advocates, clinical research, patient-centric research

## Abstract

Objectives: Good Clinical Practices (GCP) are essential for patient-centric research. The standard bioethics and GCP training emphasizing a “one-size-fits-all” approach may not adequately equip ethics committee members, especially the lay and social scientist members, towards their critical role in reviewing clinical trials and related documentation. This article explores a patient-centered, patient advocates-driven training program focused on raising awareness about research ethics and GCP among patients, advocates and ethics committee members.

Methods: A patient advocates-driven program called Patient Advocates for Clinical Research (PACER) conducted trainings focused on GCP for patient-centric research for patients, advocates and ethics committee members. Pre- and post-workshop questionnaires were used to assess the participants’ knowledge of GCP.

Results: The workshop was attended by 116 participants. Of these 91 consented to participate in questionnaire evaluation that assessed participants’ knowledge on ethics committee (EC) functionality, research ethics and data confidentiality. Pre-workshop evaluations highlighted knowledge gaps. Only 16.5% were familiar with the primary ethical consideration for vulnerable populations and 69.2% were knowledgeable about data governance. Post-workshop evaluations demonstrated significant overall response improvement of 5.4% (𝜒^2^=13.890; p<0.001). The understanding of ethical considerations for vulnerable populations rose by 15.4% (p=0.007), and knowledge of data privacy regulations improved by 11.0% (p=0.041).

Conclusion: The workshop under PACER initiative highlighted the knowledge gaps in understanding the EC functionality, research ethics and data confidentiality. The workshop effectively fostered participants' understanding of ethical research practices.

## Introduction

Clinical trials are an integral part of medical progress, rigorously striving to develop new treatments and improve patient outcomes. The increasing complexities and cost of process-based clinical research have contributed to the shift towards personalized care and patient involvement. Just as the old saying “Salus populi suprema lex esto” translates to “The welfare of the people should be the supreme law”, the patient-centric approach prioritizes the patient’s well-being [1]. Implementation of this approach requires ensuring the quality, efficiency, and effectiveness of patient-centric trials, along with critical reevaluation of current training practices. Patient-centered research engages patients in identifying unmet needs and refining the design and conduct of clinical studies, as well as advising on subsequent regulatory assessments and post-marketing vigilance [2]. Good Clinical Practices (GCP) serves as the backbone of ethical clinical research governing the design, conduct, recording, reporting, and monitoring of clinical trials involving human participants. The International Council for Harmonisation of Technical Requirements for Pharmaceuticals for Human Use (ICH) established GCP Guideline (ICH-E6 R2) as the global standard prioritizing the rights, safety and well-being of research participants while ensuring credibility of data generated from clinical trials [3]. The coronavirus disease 2019 (COVID-19) pandemic spurred the use of remote elements in clinical trials, traditionally conducted on-site, raised concerns about the compatibility of digital innovations with existing GCP guidelines. The revised draft ICH GCP E6(R3), endorsed in May 2023, encouraged innovation for efficiency and also emphasized incorporating patient needs and preferences in clinical trials [4].

Institutional Ethics Committees (IECs) are independent bodies within research institutions responsible for reviewing and approving clinical trial protocols before they commence. The IECs play a vital role in assessing the ethical acceptability of the research, ensuring its compliance with GCP guidelines and protecting participants' rights [5]. Training IEC members in GCP is crucial for equipping them with a comprehensive understanding of ICH-E6 principles, research ethics frameworks, and regulations governing clinical trials to effectively fulfill their responsibilities [6].

The traditional "one-size-fits-all" approach to research ethics and GCP training, primarily focused on investigators and sponsors, might not be sufficient for the complexities of modern research. The Clinical Trials Transformation Initiative (CTTI) identified informed consent, compliance with protocol and protection of participants’ health and safety as the three crucial tasks for quality conduct of clinical trials. The study also highlighted that the one-size-fits-all GCP guidelines ensured ethical standards, however redundancy and lack of real-world search made them uninspiring. The takeaways from the CTTI project focused on fit-for-purpose, practical skills, hands-on application, mentorship and more engaging approaches as facilitators for improving GCP training [7,8].

Current regulations and protocols rarely mandate a focus on patient needs, potentially contributing to health disparities among trial participants. Addressing these issues requires a multi-pronged approach, including broader systemic changes but also a shift in training that empowers stakeholders to prioritize patient perspectives [9]. The inclusion of patients and advocates throughout the research process holds immense potential. Their unique experiences, insights, and advocacy efforts can significantly improve research design and implementation [10]. This can be achieved by fostering an environment of active listening and collaboration.

Traditional research approaches often overlook the perspectives and experiences of those most affected by health inequities. Community-Based Participatory Research (CBPR) offers an alternative by directly involving community members throughout the research process. CBPR builds trust and empowers communities to become active partners in identifying their health concerns, designing research studies, and advocating for policy changes. This collaborative approach aims to generate relevant research and equip communities with the knowledge and skills necessary to effectively advocate for policies that address the root causes of health disparities and ultimately achieve health equity [11].

The emphasis on patient-centric research represents a critical shift in the way clinical trials are conducted. By prioritizing patient empowerment, ensuring access to quality information, and fostering collaboration between all stakeholders, this approach holds the potential to revolutionize healthcare delivery and improve patient outcomes on a global scale. Training and education of not only the investigators but all the stakeholders are crucial for developing new strategies in target-specific patient-centric research to improve the conduct of quality clinical trials, as well as trial efficiency and effectiveness. However, addressing training gaps, promoting information quality and accessibility, and acknowledging the broader context of health disparities will be crucial for the success of this new paradigm in clinical research. Here, we discuss a program for patient-centric research driven by patient advocates aimed to educate patients, advocates, navigators and ethics committee members about research ethics and patient-centric GCP.

## Materials and methods

A two-tiered education program, Patient Advocates for Clinical Research (PACER), comprising a self-paced online learning series followed by in-person workshops was implemented pan-India between January 2023 to January 2024 (Appendix 1-2) [12]. Institutional Ethics Committee of CanKids KidsCan (IEC-CK-2023-03) approved the study. The questionnaire was self-responded with implied consent from the participants, as the study involved minimal risk and the anonymized collection of data. The PACER workshop was focused on Bioethics and Good Clinical Practice for patients and ethics committee members. In this cross-sectional study, anonymized and self-reported data using pre- and post-structured questionnaires was collected before and after the workshop.

Population

IEC members, patients, patient advocacy groups (PAGs) and patient navigators (PN) working in areas of cancer (adult and paediatric), multiple myeloma, dermatological diseases, blood-related disorders, cardiovascular diseases, type I diabetes, spinal muscular atrophy, inflammatory bowel diseases, clinical research, palliative care, and supportive services were included. The recruitment of participants was done by sending email invitations to 25 IEC institutions and 50 PAGs and/or non-government organizations (NGOs). The email addresses were obtained from PAG coordinators, and follow-up emails were sent to the non-responders. Around 51 IEC members, 35 PNs and 30 patient advocates responded to the email invites. The enrolment of the participants in the study required at least higher secondary education and attended the PACER program. Among 116 enrolled participants, 91 of them gave implied consent to participate in the questionnaire-based survey.

Intervention

The orientation program educated participants through interactive sessions addressing the following topics - a) Ethics Committee and role of layperson/patient advocates in research; b) Experience of layperson in review; c) Laws and regulation; and d) Reviewing consent forms.

Comparison

Assessment of participants understanding through evaluation of pre- and post-workshop questionnaires (Appendix 3-4) each comprising nine multiple-choice questions on EC functionality and data governance. Before the initiation of the study, the questionnaire was pilot-tested on five respondents. Feedback was collected to refine the questions for clarity, relevance and comprehensiveness. The questionnaire was provided to 91 participants who had consented to undertake the survey.

Outcomes

Assessment of awareness levels and knowledge improvement will give an insight into the impact of the program on participants’ knowledge gain. Improvement in knowledge was measured by comparing participants' scores on pre- and post-workshop questionnaires. Scores were calculated by assigning numerical values to correct answers and summing them up for each participant.

Statistical analysis

Pre- and post-questionnaire responses for each question are presented as frequencies and percentages. An absolute improvement for each question and an overall improvement are presented as frequencies and percentages. The statistical analysis for improvement in participants’ answer correctly across various questions was performed by McNemar test. This statistical test is well-suited for analyzing paired categorical data, often used in pre-post or before-after studies like this one. A significance level of p < 0.05 was applied. SPSS version 26.0 (IBM Corp., Armonk, NY, USA) was used for data analysis.

## Results

The workshop was held on 29th January 2024 and was attended by 116 participants. The average age of participants was 41.8 ± 13.2 (Range: 20 - 75) years. Female preponderance (74/116; i.e. 64.7%) was seen among the attending participants. A high participation rate was seen in the 31 - 50 years age range with 68 participants out of 116 (58.6%) belonged to this group (Table 1).

**Table 1:** Demographic details of participants attending the Patient Advocates for Clinical Research (PACER) workshop 4 – Orientation program towards patient-centric research Gender-based and age-based distribution represented as frequencies and percentages. The average age of participants represented as mean ± standard deviation.

	Number of Participants (n = 116)	Percent (%)
Gender		
Female	74	64.7%
Male	41	35.3%
Age (Years)		
< 20	2	1.7%
21 - 30	22	19.0%
31 - 40	34	29.3%
41 - 50	34	29.3%
51 - 60	10	8.6%
> 60	14	12.1%
Mean ± SD (Range)	41.8 ± 13.2 (20 - 75)	

Participants were assessed for their knowledge about PACER, ethical framework in clinical research and regulatory guidelines for data management in clinical research through questionnaire evaluation. Out of 116 participants, 91 agreed to participate in the questionnaire-based evaluation. During pre-evaluation, 64/91 (70.3%) participants were aware of ‘PACER’ which raised to 67/91 (73.6%) (p=0.549) participants after the workshop. When questioned about ‘their preference for training’, 43/91 (47.3%) opined for both in-person workshops and self-paced online training during pre-evaluation and that rose to 47/91 (51.6%) in post-evaluation (Table 2).

**Table 2:** Pre- and post-questionnaire response evaluation of participants attending the Patient Advocates for Clinical Research (PACER) workshop 4 – Orientation program towards patient-centric research Pre- and post-questionnaire were attempted by 91 out of 116 participants. The correct responses have been represented in bold. The distribution of responses are represented as represented as frequencies and percentages.

Questions	Options	Pre-workshop (n = 91)		Post-workshop (n = 91)	
1. PACER (Full Form)?	Patient Advocates for Clinical Research Engineers and Report Frameworks	20	22.0%	23	25.3%
	Patient Advocates for Clinical Research	64	70.3%	67	73.6%
	Public Advocates for Clinical Reports Empowerment and Regulatory Frameworks	3	3.3%	1	1.1%
	Patient Advocates for Clinical Research Engagement and Reports Frameworks	3	3.3%	0	0.0%
	I don’t know	1	1.1%	0	0.0%
2. What is your personal preference for training	In person Workshops	24	26.4%	25	27.5%
	Self-paced online training	24	26.4%	19	20.9%
	Both	43	47.3%	47	51.6%
3. What is the primary responsibility of an Ethics Committee member	Careful Data analysis	7	7.7%	6	6.6%
	Participant recruitment strategy	3	3.3%	6	6.6%
	Ensuring ethical standards	77	84.6%	78	85.7%
	Budget management	1	1.1%	0	0.0%
	Attending Meeting	3	3.3%	1	1.1%
	I don’t know	0	0.0%	0	0.0%
4. What role does an Ethics Committee member play in monitoring ongoing research studies?	Authoring research papers	6	6.6%	4	4.4%
	Conducting experiments	3	3.3%	1	1.1%
	Ensuring ongoing ethical compliance	82	90.1%	84	92.3%
	Recruiting new participants	0	0.0%	0	0.0%
	I don’t know	0	0.0%	2	2.2%
5. According to you which sentence is most relevant to the Layperson of an Ethics Committee	Layperson represents the local community or its concerns	20	22.0%	14	15.4%
	Lay person has the viewpoint of someone who was going to be a subject of the study	9	9.9%	12	13.2%
	Lay person reviews scientific protocol	3	3.3%	0	0.0%
	Both a and b	56	61.5%	62	68.1%
	I don’t know	3	3.3%	3	3.3%
6. In the context of research ethics, what does "informed consent" refer to?	Participants' agreement without understanding	3	3.3%	0	0.0%
	Participants' voluntary and knowledgeable agreement	85	93.4%	87	95.6%
	Anonymous participation	2	2.2%	3	3.3%
	I don’t know	1	1.1%	1	1.1%
7. Which ethical consideration is most relevant when evaluating the inclusion of vulnerable populations in a research study?	Autonomy	15	16.5%	29	31.9%
	Beneficence	40	44.0%	31	34.1%
	Nonmaleficence	26	28.6%	25	27.5%
	I don’t know	10	11.0%	6	6.6%
8. If there is a potential ethical violation, what is the appropriate course of action for an Ethics Committee?	Ignore the issue	0	0.0%	0	0.0%
	Investigate the matter independently	13	14.3%	11	12.1%
	Consult with legal authorities	13	14.3%	12	13.2%
	Initiate a formal review and report appropriately.	64	70.3%	65	71.4%
	I don’t know	1	1.1%	3	3.3%
9. What is the primary purpose of the Digital Personal Data Protection Act 2023?	Facilitating data breaches	9	9.9%	7	7.7%
	Regulating the use of personal data	63	69.2%	73	80.2%
	Encouraging unrestricted data sharing	7	7.7%	2	2.2%
	To stop use of data	2	2.2%	1	1.1%
	I don’t know	10	11.0%	8	8.8%

Further, participants’ knowledge about the roles and responsibilities of EC members in clinical research/trial was assessed. During initial evaluation, participants when asked about ‘the primary responsibilities of an ethics committee member’, 77/91 (84.6%) answered correctly as ‘ensuring ethical standards’ that raised to 78/91 (85.7%) during post-evaluation (response improvement=1.1%; p=1.000). Similar increase in participants’ post-evaluation responses was seen in comparison to pre-evaluation when questioned ‘what role does an ethics committee member play in monitoring ongoing research studies’ (improvement=2.2%; p=0.727). During pre-evaluation, participants when asked ‘according to you which sentence is most relevant to the layperson of an ethics committee’, 56/91 (61.5%) participants were aware that ‘layperson represents the local community or its concern and has the viewpoint of someone who was going to be a subject of the study’. The awareness of the role of layperson in EC improved by 6.6% (p=0.345) during post-evaluation where 62/91 (68.1%) opined for the correct response.

Pre-evaluation on research ethics for participants regarding ‘what does informed consent refer to’, 85/91 (93.4%) participants correctly answered as ‘participants’ voluntary and knowledgeable agreement’. Meanwhile, 87/91 (95.6%) participants responded correctly during post-evaluation (response improvement=2.2%; p=0.727). Participants when asked ‘which ethical consideration is most relevant when evaluating the inclusion of vulnerable populations in a research study’, only 15/91 (16.5%) correctly answered ‘autonomy’. However, a response improvement of 15.4% (p=0.007) was seen during post-workshop evaluation where 29/91 (31.9%) participants opted for the correct answer. Participants when asked ‘if there is a potential ethical violation, what is the appropriate course of action for an ethics committee’, 64/91 (70.3%) answered correctly ‘initiate a formal review and report appropriately’ during pre-evaluation. During post-evaluation, 65/91 (71.4%) participants opined for the correct response (response improvement=1.1%; p=1.000) (Table 2).

Data handling and confidentiality being an important aspect of clinical research, participants were questioned ‘what is the primary purpose of the digital personal data protection 2023?’. During initial evaluation, 63/91 (69.2%) participants correctly responded ‘regulating the use of personal data’. During post-evaluation, 73/91 (80.2%) participants responded with the correct answer showing a response improvement of 11.0% (p=0.041) (Table 2).

The post-workshop evaluation of participants’ responses showed an overall improvement of 5.4% (𝜒2=13.890; p<0.001) which was highly significant in comparison to the pre-workshop evaluation. 

## Discussion

This study evaluates the understanding of stakeholders about GCP for patient-centric research and the impact of a PAG-driven patient-centric training program. The pre-workshop evaluation highlighted the knowledge gaps in EC functionality, research ethics and data confidentiality. However, a substantial improvement of 5.4% in participants' knowledge was noted across all assessed areas. The findings of the study suggest PACER workshop effectively addressed the knowledge gaps thereby strengthening an understanding of patient-centric ethical research practices among a diverse group of potential clinical research stakeholders. Thus, the PACER initiative represents a novel approach that seeks to empower patients as active participants and stakeholders in the research prioritizing individualized care and patient-centered outcomes.

A transition towards a patient-centric approach requires investigators, research stakeholders and EC members to be well-informed with the necessary skills and knowledge to effectively collaborate with patients and advocates. CTTI recommends expansion of traditional "one-size-fits-all" GCP training to a more targeted approach identifying potential execution risks and focused training. The recommendations emphasize on the necessity of designing educational programs to engage adult learners and address the identified knowledge gaps and specific trial needs. Additionally, CTTI suggests implementation of active learning methods like mentoring and simulations as valuable tools to promote stakeholder engagement in patient-centric research. In addition to tailored educational programs, resources like CTTI’s Quality by Design [13], Investigator Community [14], Investigator Qualification Framework [15], a Quick Reference Guide to Documenting Qualification for Investigators and Their Delegates [16], and a documentation template [15] should be utilized for developing protocols, recruiting qualified research team and conducting quality clinical trials [17]. The PACER initiative can play a crucial role in promoting more effective methodologies for adult learning, ensuring investigators are qualified to conduct high-quality clinical trials while prioritizing patient safety and data integrity.

Organizations like the Patient-Centered Outcomes Research Institute in the USA [18] and the James Lind Alliance in the UK [19] empower patients by educating them, identifying their priorities, designing patient-centric studies, availing access to trials and evaluating patient experiences. The National Cancer Institute (NCI) initiative National Clinical Trials Network (NCTN) proposed a framework to involve patients early on in research design and protocol development, establishing patient advisory committees for continuous input, and utilizing accessible communication strategies to reach diverse patient populations. Additionally, the framework also encouraged active patient engagement in recruitment drives and trial conduct itself. Implementation of this multifaceted approach, researchers can leverage patient perspectives to design more relevant and patient-centered cancer clinical trials, ultimately accelerating progress in cancer research and improving patient care [20]. The multimodal methodology of the PACER initiative raised awareness of participants’ rights in clinical research amongst patients, caregivers and advocates. The initiative introduced the concept of ‘expert’ patient to patients and advocates thus marking a shift of the research landscape closer to patient-centric research [21].

The emergence of India as a global hub for clinical trials stems from a confluence of favorable conditions like a heterogeneous patient population, experienced investigators, affordable research infrastructure, and well-defined regulatory guidelines [22]. The burgeoning demands of clinical research dynamics have also brought concerns about data integrity, accuracy and ethical conduct. The lacunae were permeated through the launch of the National Patient Safety Implementation Framework 2018-2025 (NPSIF) by the India Ministry of Health and Family Welfare through strengthening of patient safety and quality of healthcare. This framework was integrated with the National Health Mission, Clinical Establishment Act and Pharmacovigilance Program to encourage and consolidate patient safety research [23]. NPSIF in association with non-profit healthcare organizations, patient rights groups and patient safety organizations directed initiatives to improve patient networking for patient centricity, create awareness and aid stakeholders’ collaboration for developing patient safety policies in India [24]. The collaborative effort of NPSIF with medical, nursing and pharmacy programs to integrate the World Health Organization’s (WHO) Patient Safety Curriculum in education program will equip future healthcare professionals to prioritize patient safety [24]. Focus group discussions study amongst medical professionals in Kerala identified resource constraints, poor health care delivery systems, hierarchical work culture, inadequate provider training and patient education/awareness [25]. Similarly, an online knowledge and awareness assessment about clinical research functions based on the New Drugs and Clinical Trials (NDCT) Rules 2019 among researcher, research staff (RS) and EC members identified knowledge gaps in informed consent (IC) process, EC monitoring of ongoing trials and post-trials access [26]. In our study, the knowledge assessment among patients, advocates and EC members identified gaps in the IC process (16.5%) and data governance (69.2%), implying the need to develop informed consent and data governance training programs tailored for EC members and patients.

Accurate and understandable materials promote trust among patients and facilitate them to make informed decisions about their participation in research [27]. Social media platforms have proved as an important tool in dissemination of research-based content, patient education and engagement. The health literacy among patients has improved with availability of educational content in various formats, from disease awareness videos to patient experience blogs. The online communities on social media also encourage patients in finding peer support, sharing experiences, and combat feelings of isolation [28]. Social media has encouraged active participation and thoughtful communication among diverse audiences thus, empowering patients to make informed decisions about their health. Overall, availability of information on social media platforms has improved patients’ access to health, empowered them with educational content and promote active participation in their healthcare journey [28]. However, critical evaluation for the quality and accuracy of content is necessary to navigate any potential misinformation [29]. PACER's focus on patient engagement creates a need for readily available, trustworthy information on clinical research.

Limitations of the study

The study highlighted the knowledge gaps in GCP training, however the study has some limitations. Firstly, the selection bias does not represent the perspectives of all relevant clinical research stakeholders. The participant’s interest in clinical research may influence their baseline knowledge and responses, hence selection bias. Secondly, the knowledge assessment is self-reported and may not reflect the actual understanding of the participants. The participants’ memory or interpretation of information might influence their responses. Arrangement of training programs across various regions and sequential follow-ups with the participants faces financial limitations. Successful stakeholder engagement necessitates hands-on training of the stakeholders and ensuring proper implementation. Incorporation of feedback helps in understanding the ever-changing research landscape and continuous evaluation of training programs. Lastly, the focus of the workshop was aimed at knowledge gain and did not emphasize on development of practical skills required for implementation of GCP principles in real-world research settings.

## Conclusions

The PACER program highlighted the key challenges presented by standard GCP “one-size-fits-all” approach by foregrounding the needs of patients. The program focused on EC functionality, research ethics and participants rights to strengthen the understanding of patients, advocates and navigators for fostering informed participation. The emphasize on critical areas like vulnerable population protection and data governance served as a stepping stone to improve the comprehension of EC members, particularly layperson and social science representatives. The overall knowledge improvement (5%) post-workshop was statistically significant. However, the knowledge gaps in understanding the role of EC members, participants vulnerability and data confidentiality were eminently accentuated. This raises the necessity for organizing such tailored GCP trainings to empower diverse stakeholders for ethical research. The success of PACER shall lie in its ability to initiate collaboration between patients, researchers, and healthcare professionals, creating a win-win research environment for both patients and the scientific fraternity.

## Figures and Tables

**Figure 1:**
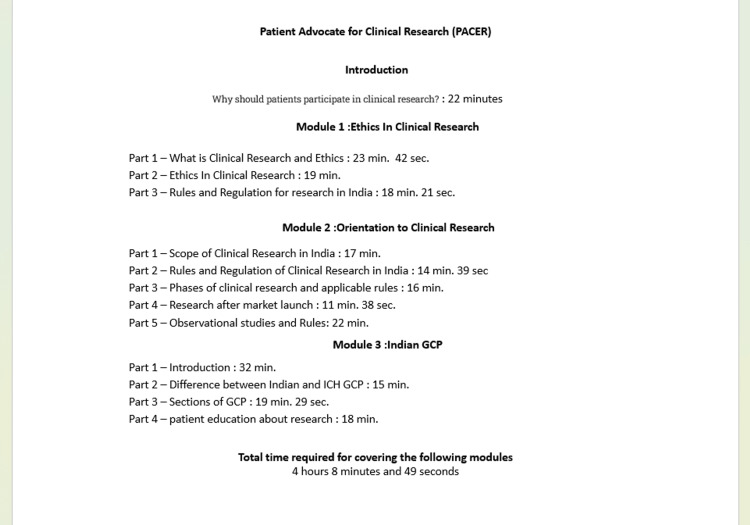
Detail description of the online learning series PACER: Patient Advocates for Clinical Research, GCP: Good Clinical Practices, ICH: International Council for Harmonisation of Technical Requirements for Pharmaceuticals for Human Use

**Figure 2:**
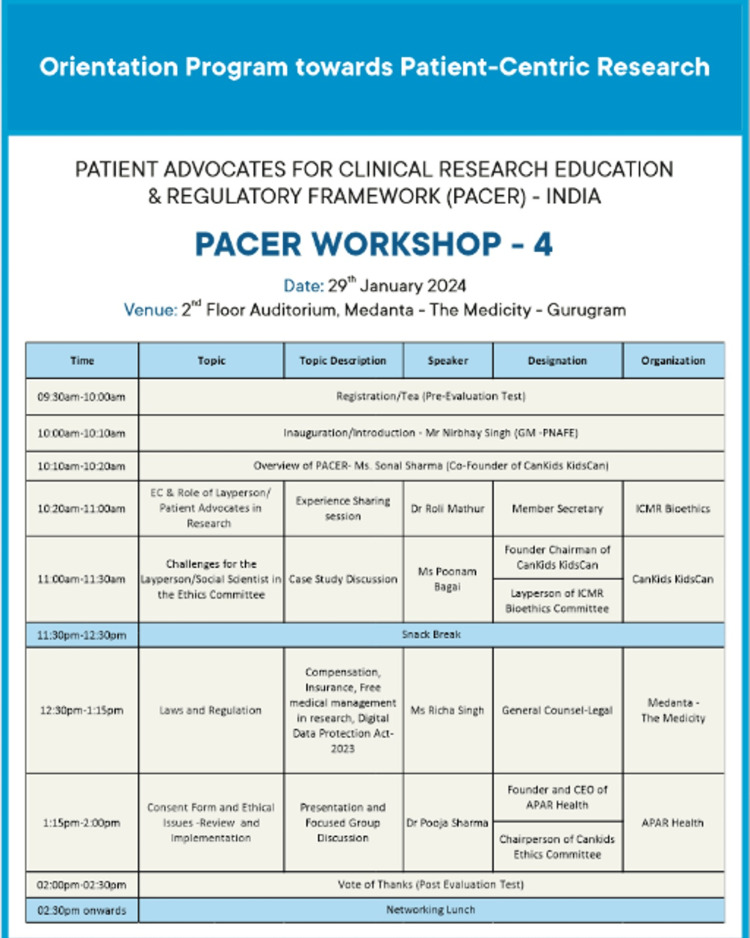
Detailed description of Patient Advocates for Clinical Research (PACER) Workshop 4- Orientation Program towards Patient-Centric Research

**Figure 3:**
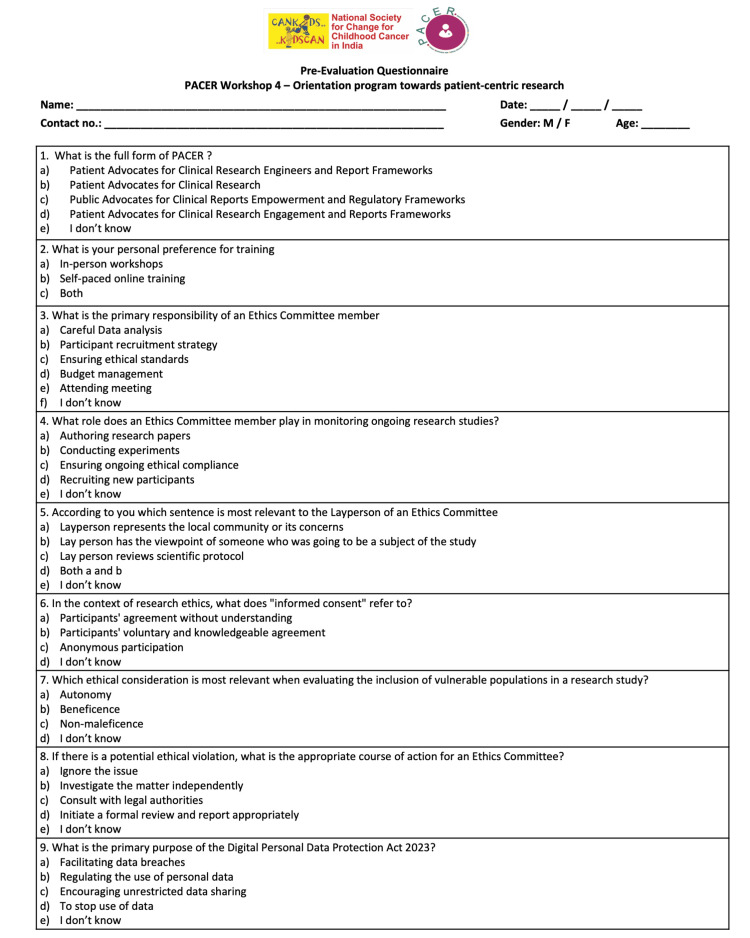
Pre-evaluation Questionnaire, Patient Advocates for Clinical Research (PACER) Workshop 4: Orientation program towards Patient-Centric Research

**Figure 4:**
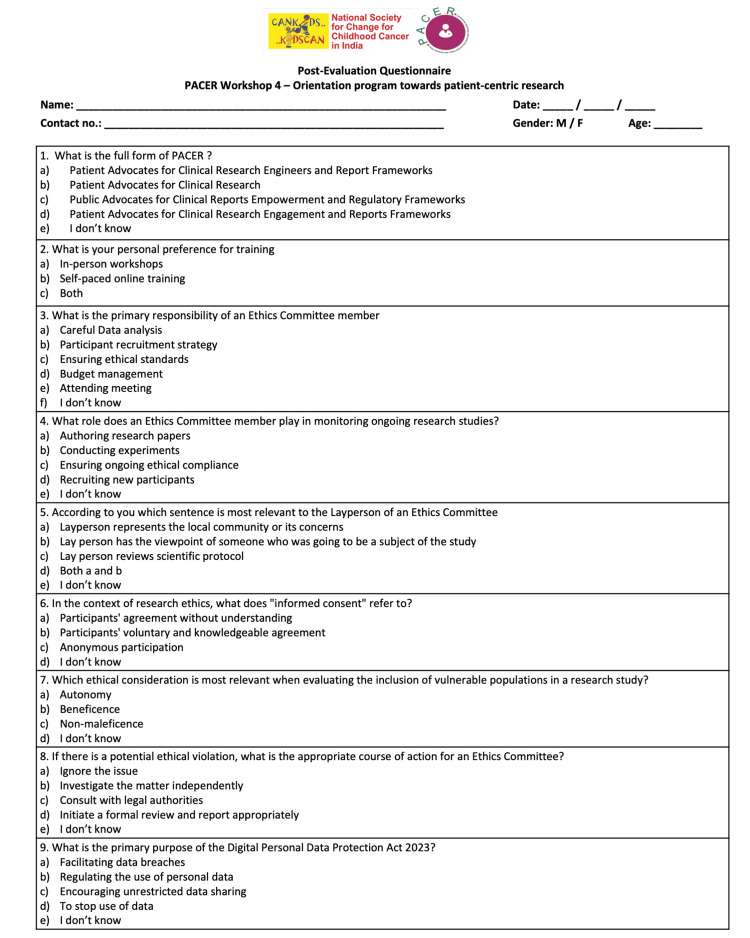
Post-evaluation Questionnaire, Patient Advocates for Clinical Research (PACER) Workshop 4: Orientation program towards Patient-Centric Research

## Appendices

Appendix 1

Appendix 2

Appendix 3

 Appendix 4
